# A Randomised Controlled Trial Comparing the Therapeutic Efficacy and Safety of Oral Cyclosporine and Oral Betamethasone Minipulse Therapy in the Treatment of Alopecia Areata

**DOI:** 10.7759/cureus.84005

**Published:** 2025-05-13

**Authors:** Shibashis Chatterjee, Hemanta K Kar, Suvigya Sachan, Laxman Besra, Mitanjali Sethy

**Affiliations:** 1 Dermatology, Venereology, and Leprosy, Shri Ramkrishna Institute of Medical Sciences and Sanaka Hospitals, Durgapur, IND; 2 Dermatology, Venereology, and Leprosy, Kalinga Institute of Medical Sciences, Bhubaneswar, IND; 3 Dermatology, Venereology, and Leprosy, All India Institute of Medical Sciences, Mangalagiri, Mangalagiri, IND

**Keywords:** alopecia areata, betamethasone, cyclosporine, oral mini-pulse, pulse drug therapy

## Abstract

Background

Multiple systemic agents have been evaluated for their efficacy and safety in alopecia areata (AA); however, there is a paucity of comparative studies available in literature.

Objective

To compare the efficacy and safety of oral cyclosporine and betamethasone mini-pulse therapy in the treatment of alopecia areata (AA).

Materials and methods

This non-blinded, randomized, parallel-group controlled trial included 60 patients. Group A (n=30) received oral cyclosporine 3 mg/kg body weight, and Group B (n=30) received oral betamethasone mini-pulse therapy in a dose of 0.1mg/kg body weight on two consecutive days per week for 12 weeks each.

Results

In the cyclosporine group, 53.3% of the patients responded to the treatment as compared to 33.3% of the patients in the betamethasone mini-pulse therapy group. In the cyclosporine group, patients with severe AA were found to respond better to the treatment. Based on the patient self-assessments, 73.3% of patients in the cyclosporine group and 43.3% of patients in the betamethasone mini-pulse group rated their hair regrowth as excellent or good. Adverse effects to the therapy were noted in 76.6% of patients in the cyclosporine group, whereas it was 53.3% in the betamethasone minipulse group. All of them were mild and reversible in nature.

Conclusion

Oral cyclosporine therapy appeared to be superior to betamethasone mini-pulse therapy in terms of therapeutic efficacy.

## Introduction

Generally described as a recurrent non-scarring type of hair loss, alopecia areata (AA) can affect any hair-bearing area. Clinically, AA can manifest in many different patterns. Even though it is a medically benign condition, it can cause humongous emotional and psychosocial distress in affected patients. About 1.7% of the population experiences an episode of alopecia areata during their lifetime. The prevalence of alopecia areata in the general population is 0.1-0.2% with no sex predilection. It mostly affects children and young adults, although any age group may be affected [1]. Though it is a self-limiting condition and many of these cases of alopecia areata tend to regress spontaneously within 1-2 years, most of the patients are anxious about the condition as it causes severe cosmetic disfiguration, and due to the stigmata associated with it in society. Hence, it causes a significant impact on the quality of life, and patients are eager for a quicker treatment of the condition.

Current treatment modalities available are corticosteroids (topical, intralesional, oral) [2], oral cyclosporine [3], tacrolimus [1], minoxidil [4], contact immunotherapies [5] like squaric acid dibutyl ester, diphencyprone [6], dinitrochlorobenzene and photo(chemo)therapy using UVA and psoralen [7,8]. Systemic agents like corticosteroids, when administered daily,y may lead to various adverse effects like hyperglycaemia, osteoporosis, cataracts, glaucoma, immunosuppression, obesity, acne, and Cushing syndrome. To minimize the adverse effects of systemic corticosteroids, oral mini-pulse therapy was introduced. Earlier betamethasone mini-pulse therapy has been shown to be equally efficacious with fewer adverse effects as compared to daily therapy and is still regarded as a relatively safe and effective therapeutic option for the treatment of AA. Cyclosporine has a therapeutic benefit in AA by reducing perifollicular lymphocytic infiltrates [1].

The present study is designed with an aim to compare two modalities, i.e., oral cyclosporine versus oral betamethasone mini-pulse therapy, in the treatment of alopecia areata (AA) and to assess their comparative treatment effectiveness and safety profiles.

## Materials and methods

In this parallel-group, randomised, controlled, non-blinded study, patients suffering from alopecia areata who visited the outpatient department of dermatology, venereology, and leprosy of our hospital between January 2019 and June 2020 were enrolled.

Ethical considerations

The Institutional Ethics Committee of Kalinga Institute of Medical Sciences approved the study (Ref: KIMS/KIIT/IEC/114/2018, Dated 07.09.2018). The study was registered under Clinical Trials Registry - India (CTRI) (CTRI Reg No. -CTRI/2019/01/017202).

Sample size

**Figure 1:**
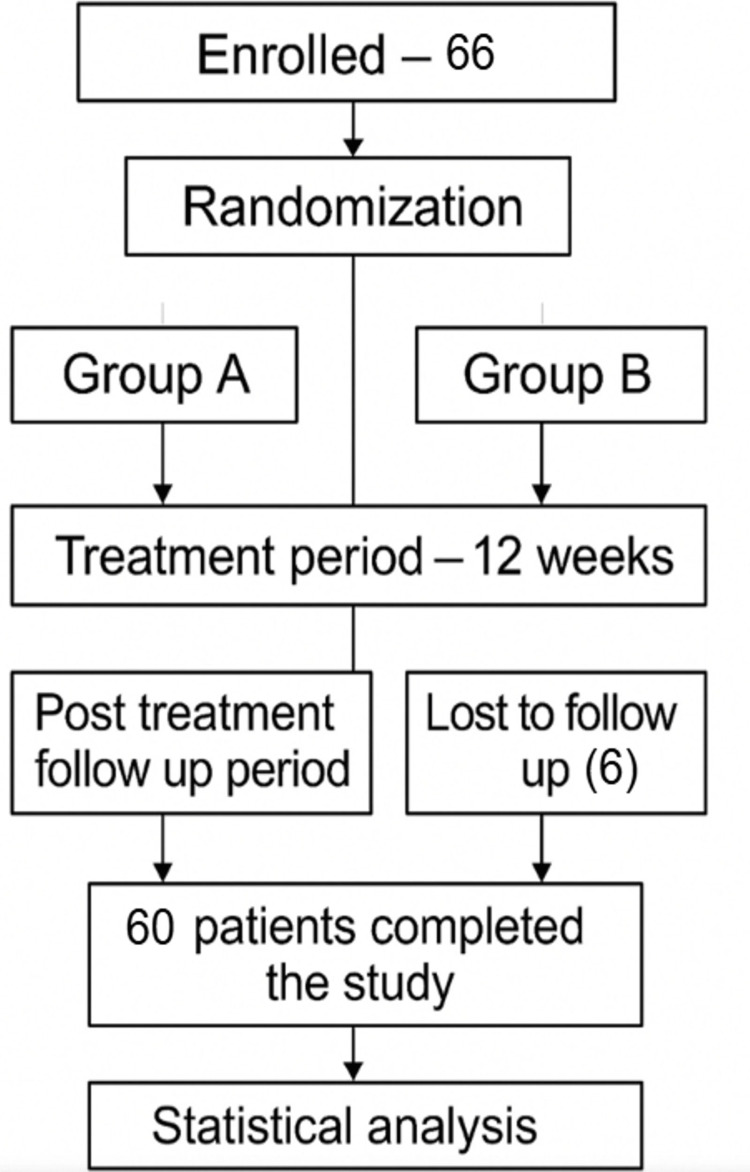
CONSORT diagram outlining the flow of selection of participants through the study. CONSORT: CONsolidated Standards Of Reporting Trials

The required sample size was calculated to be 60 (30 for each group) based on an assumed standard deviation of 1.0, an expected difference of 0.60, a 5% significance level, and 80% power. To account for potential dropouts, an additional six cases were included in the study (Figure 1).

Procedure of study

A total of 66 patients diagnosed with alopecia areata fulfiling the inclusion and exclusion criteria, presenting to the dermatology department of our tertiary care hospital during the study period, were included in the study. The diagnosis of alopecia areata (AA) was made on clinical grounds and dermoscopy. The clinical diagnosis was based on the presenting features of an asymptomatic, circumscribed, smooth, non-scaly, non-scarring patch of hair loss on the scalp and other hair-bearing areas of the body. Those having <50% scalp hair loss on the scalp were considered to have mild AA, and those with ≥50% scalp hair loss, alopecia totalis (AT), and alopecia universalis (AU) were considered to be severe AA. In doubtful cases, fungal scraping from the patch was done to rule out tinea capitis. A thorough history was elicited with regard to duration of symptoms, progression of disease, history of past episodes, treatment history, personal and family history of atopy, diabetes mellitus, hypertension, and history of any other autoimmune diseases.

A meticulous general and systemic examination was performed, including ophthalmological evaluation. A complete dermatological examination was done to note any other associated dermatoses. Nail changes and mucosal changes were recorded.

The location and size of the patches of AA, presence of hair, including exclamation mark hair, coudability sign, and pluckability of hair at the margins were all recorded. Baseline investigations, including complete hemogram, blood sugar, serum electrolytes, renal and liver function tests, and urinalysis, were conducted for each AA patient. Sixty-six consecutive cases fulfilling the inclusion criteria were enrolled for this therapeutic study.

Inclusion and exclusion criteria

Patients between 8-60 years of age diagnosed with alopecia areata who had not undergone any other modalities of treatment, having a patch of size >3 cm in diameter with symptoms of 3 months or more duration, were included in the study. However, several categories of patients, like those unwilling to undergo follow-up, pregnant and lactating females, individuals with renal, hepatic, or hematological disorders, patients with malignancies, patients with autoimmune diseases, patients suffering from any kind of infections and those having any kind of contraindications to the drugs used in the study were excluded.

Study design

A total of 66 patients were enrolled in this study after providing written informed consent. For participants under 18 years of age, guardian consent was also obtained. Participants were randomly allocated to one of the two treatment groups - Group A (oral cyclosporine) and Group B (oral betamethasone mini-pulse therapy) - using a computer-generated random number sequence. This sequence was generated by an independent researcher not involved in the direct management of the patients. To ensure a balanced distribution of participants in each group throughout the enrollment process, block randomisation with a pre-determined block size was employed. The specific block size was not disclosed to the investigators to further minimize the risk of predictability. To maintain allocation concealment, the treatment assignment for each participant, corresponding to their position in the randomization sequence, was placed in a sequentially numbered, sealed opaque envelope by an individual not involved in participant recruitment or assessment. These envelopes were stored securely and were opened in strict numerical order only after a participant had provided informed consent and was confirmed eligible for the study by the principal investigator. This sequential opening of envelopes ensured that the assigned treatment remained concealed until the point of intervention.

Furthermore, after enrollment and randomization, baseline characteristics of the two treatment groups were statistically analyzed to confirm comparability and ensure that the randomization process had effectively minimized potential confounding factors.

Patients in Group A received cyclosporine 3 mg/kg body weight/day orally for 12 weeks, and patients in Group B received betamethasone oral mini-pulse 0.1 mg/kg body weight/dose, on two consecutive days per week for 12 weeks. After this intensive phase, the dose of betamethasone was tapered off by 1 mg every week and off the treatment in the next 3 to 6 weeks, depending on the initial starting dose.

Outcome measures

Patients of both groups were evaluated at monthly intervals to assess the therapeutic response and to monitor the adverse effects of the therapies. Serial clinical photographs were taken every month to assess the response (primary outcome of efficacy). Table 1 illustrates the primary outcome of efficacy, which is the grading of response to the treatment.

**Table 1:** Grading of the primary outcome of efficacy. Percentage of patch covered by hair regrowth and corresponding grading of response. Table Credits Author: Shibashis Chatterjee. Adapted from Jang et al. [9].

Percentage of patch covered by hair regrowth	Grade	Response
1-10%	A	Hair regrowth covering < 10% of the patch: Very poor response
10-49%	B	Hair regrowth occurs, but is not cosmetically acceptable: Poor response
50-89%	C	Hair regrowth is cosmetically acceptable: Fair response
90-99%	D	Hair regrowth covered almost all of the patches, but the hair was not of similar density to the surrounding hair: Good response
100%	E	Complete regrowth of hair, which is indistinguishable from the surrounding area: Excellent response

The changes on the scalp, like hair regrowth, type of hair growth (vellus hair/ pigmented normal hair or no growth), extent of hair growth, any increase in size of lesion or appearance of new lesion, were noted. Similarly, any adverse effects due to drugs in both arms, like hypertension, hypertrichosis, weight gain, headache/dizziness, facial edema, acneiform eruptions, or skin atrophy, were recorded. Results were analysed clinically, photographically, as well as statistically at the end of 12 weeks.

For the purpose of statistical analysis, comparison, and interpretation, patients with excellent, good, and fair response (E, D, and C, respectively) were considered to have a positive response to treatment, and those patients with very poor and poor response (A and B) were considered to have a negative response to treatment. Only terminal hairs were used to assess hair regrowth.

Statistical analysis

Quantitative data was presented with the help of mean and standard deviation. Comparison among the study groups was done with the help of the unpaired t-test as per the results of the normality test. Qualitative data was presented with the help of a frequency and percentage table. Association among the study groups was assessed with the help of the chi-squared test. Results were graphically represented where deemed necessary. All statistical analyses were performed using IBM SPSS-20.0 (IBM Corp., Armonk, USA), with a p-value < 0.05 considered statistically significant.

## Results

Patient characteristics

Out of 66 cases enrolled in this study, six cases were lost to follow-up (three in each group). Among the 60 study completed cases, Group A, the cyclosporine group (n=30), consisted of 17 male and 13 female patients with a mean age of 26.50 ± 10.67 years. Of the patients, four had AA for more than 12 months, three had a family history of AA, 11 had mild AA (<50% scalp hair loss), and 19 had severe AA (≥ 50% scalp hair loss, AT, and AU). Cyclosporine was given at a dose of 3 mg/kg body weight for a duration of 3 months.

Group B, the betamethasone mini-pulse therapy group (n=30), consisted of 16 male and 14 female patients with a mean age at onset of 25.73 ± 9.38 years. Of these 30 patients, three had AA for more than 12 months, two had a family history of AA, 12 had mild AA, and 18 had severe AA. Betamethasone therapy was given as oral weekly pulses at a dose of 0.1 mg/kg body weight/dose on two consecutive days per week, followed by 5 days off treatment, for 12 weeks.

Treatment response and side effects

The response rate was 16 (53.3%) in Group A and 10 (33.3%) in Group B, as illustrated in Table 2. There was no significant difference between the groups as per the chi-square test (p>0.05).

**Table 2:** Distribution of patients according to treatment response Comparison of treatment response between Group A (oral cyclosporin) and Group B (oral betamethasone mini-pulse therapy). Chi-square test was used for statistical analysis. A p-value < 0.05 was considered statistically significant.

Treatment Response	Group A		Group B		Chi-square value	p-value
							N	%	N	%
	Yes	16	53.30%	10			33.30%	2.4344	0.193
No	14	46.70%	20	66.70%					
Total	30	100%	30	100%					

**Figure 2:**
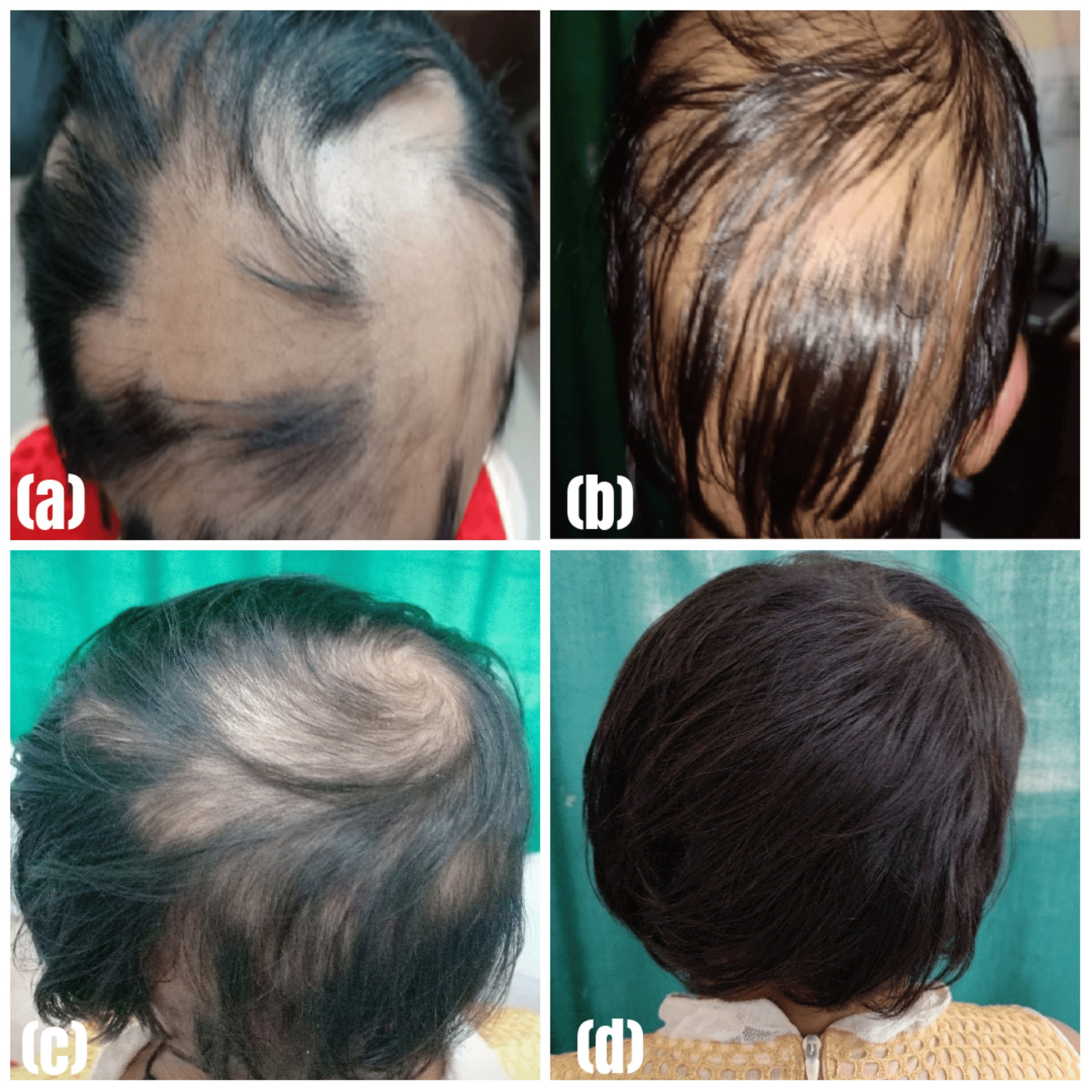
Lesions before and after treatment in Group A (cyclosporine group) (a) at first visit; (b) after 1 month; (c) after 2 months; (d) after 3 months

**Figure 3:**
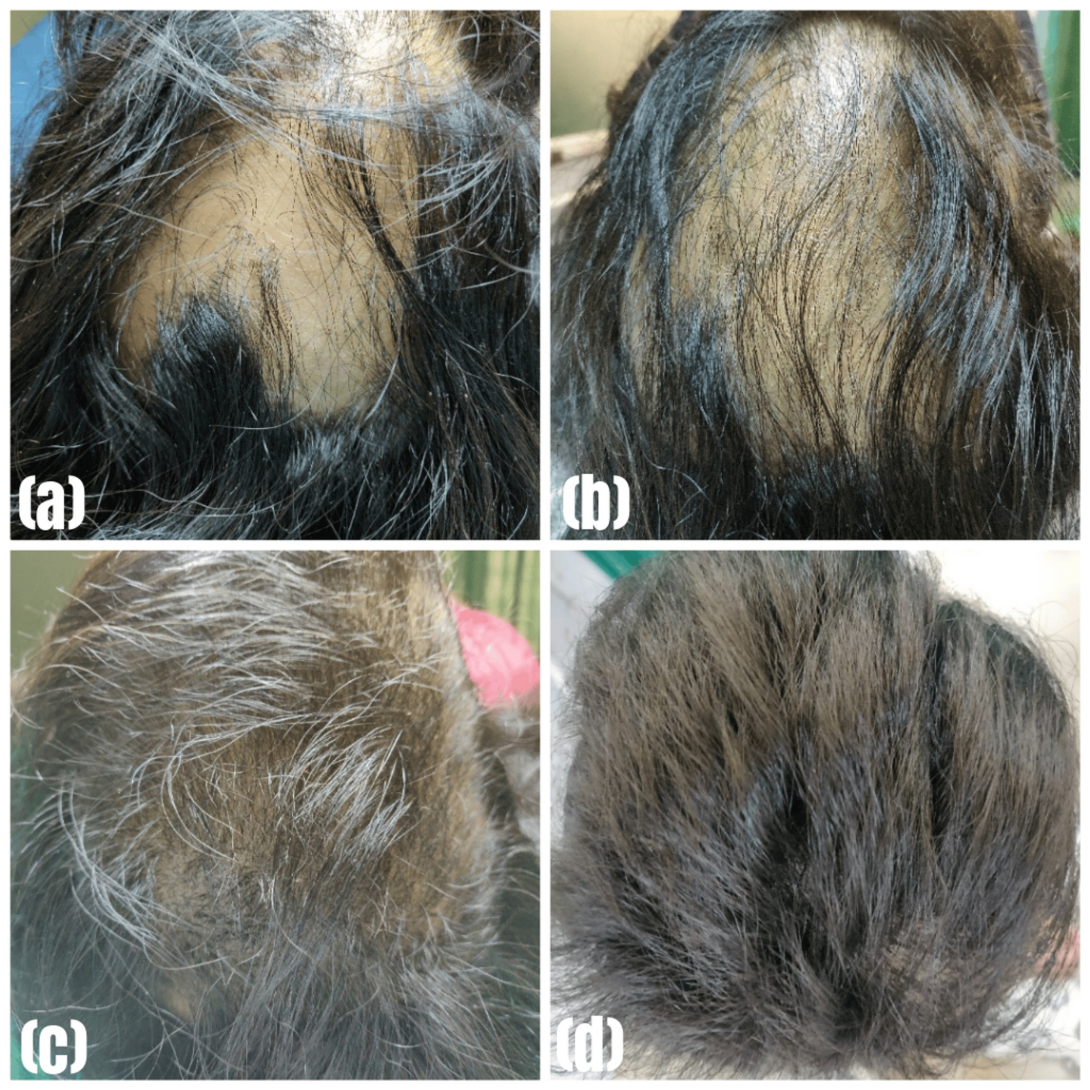
Lesions before and after treatment in Group B (betamethasone mini-pulse group) (a) at first visit; (b) after 1 month; (c) after 2 months; (d) after 3 months

Clinical improvement was documented through a comprehensive photographic assessment. Pre- and post-treatment photographs were meticulously compared, as illustrated in Figure 2a-2d and Figure 3a-3d. 

Within Group A, positive responses were seen in seven (23.3%) participants with mild hair loss and nine (30%) with severe hair loss. In Group B, seven participants (23.3%) with mild hair loss responded positively, whereas only three (10%) with severe hair loss showed a positive response. Consequently, non-response rates were 14 (46.7%) in Group A and 20 (66.7%) in Group B. In Group A, patients with severe AA responded better to the treatment than those with mild AA (30% vs. 23.3%), while in Group B, patients with mild AA responded better to the treatment than those with severe AA (23.3% vs. 10%), as illustrated in Table 3. There was no significant association of the extent of scalp hair loss with treatment response in patients in Group A, as per chi-square test (p>0.05), whereas there was a significant association of the extent of scalp hair loss with treatment response in patients in Group B, as per chi-square test (p<0.05). However, the difference between the groups in response to treatment was statistically not significant, as per chi-square test (p>0.05).

**Table 3:** Association of the extent of scalp hair loss with treatment response in patients Comparison of the association of the extent of scalp hair loss with treatment response in patients between Group A and Group B. Chi-square test was used for statistical analysis. A p-value < 0.05 was considered statistically significant.

Extent of Scalp Hair Loss		Treatment Response				Total	Chi-square value	p-value
		Yes		No				
		N	%	N	%			
Group A	Mild	7	23.30%	4	13.30%	11	1.3846	0.2393
	Severe	9	30%	10	33.40%	19		
	Total	16	53.30%	14	46.70%	30		
	Chi-square value	0.7363						
	p-value	0.3907						
Group B	Mild	7	23.30%	5	16.70%	12		
	Severe	3	10%	15	50%	18		
	Total	10	33.30%	20	66.70%	30		
	Chi-square value	5.625						
	p-value	0.0177						

As detailed in Table 4, in both Groups A and B, patients with a disease duration of 3-12 months responded better to treatment . There was no significant association between the duration of the disease and treatment response in patients in both groups as per chi-square test (p>0.05).

**Table 4:** Association of the duration of the disease with treatment response in patients Comparison of the association of the duration of the disease with treatment response in patients between Group A and Group B. Chi-square test was used for statistical analysis. A p-value < 0.05 was considered statistically significant.

Duration of Disease		Treatment Response				Total	Chi-square value	p-value
		Yes		No				
		N	%	N	%			
Group A	3-12 months	15	50%	11	36.70%	26	1.6448	0.4396
	13-24 months	1	3.30%	2	6.70%	3		
	>24 months	0	-	1	3.30%	1		
	Total	16	53.30%	14	46.70%	30		
	Chi-square value	1.8069						
	p-value	0.4052						
Group B	3-12 months	9	30%	18	60%	27		
	13-24 months	1	3.30%	1	3.30%	2		
	>24 months	0	-	1	3.30%	1		
	Total	10	33.30%	20	66.70%	30		
	Chi-square value	0.737						
	p-value	0.692						

According to the self-assessments of treatment responses, 22 (73.3%) patients in Group A rated their hair regrowth as excellent or good while eight (26.7%) rated their hair regrowth as fair or poor, whereas 13 (43.3%) patients in Group B rated their hair regrowth as excellent or good while 17 (56.7%) rated their hair regrowth as fair or poor. However, in Group A, excellent or good hair regrowth was not statistically significant as compared to that in Group B, as detailed in Table 5.

**Table 5:** Distribution of patients according to self-assessments of treatment responses Comparison of the self-assessments of treatment responses between Group A and Group B. Chi-square test was used for statistical analysis. A p-value < 0.05 was considered statistically significant.

Extent of Scalp Hair Loss		Self-assessments of Treatment Response								Total	Chi-square value	p-value
		Excellent		Good		Fair		Poor				
		N	%	N	%	N	%	N	%			
Group A	Mild	3	27.30%	6	54.50%	1	9.10%	1	9.10%	11	7.2624	0.0643
	Severe	4	21.10%	9	47.30%	2	10.50%	4	21.10%	19		
	Total	7	23.40%	15	50%	3	10%	5	16.60%	30		
Group B	Mild	2	16.60%	3	25%	4	33.40%	3	25%	12		
	Severe	1	5.50%	7	38.90%	7	38.90%	3	26.70%	18		
	Total	3	10%	10	33.30%	11	46.70%	6	20%	30		

**Figure 4:**
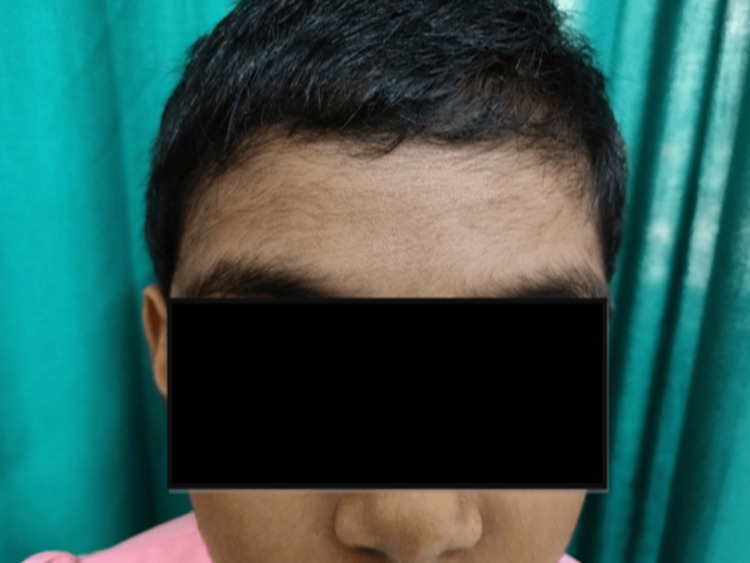
Hypertrichosis on the forehead area of an 8-years-old female due to cyclosporine therapy

**Figure 5:**
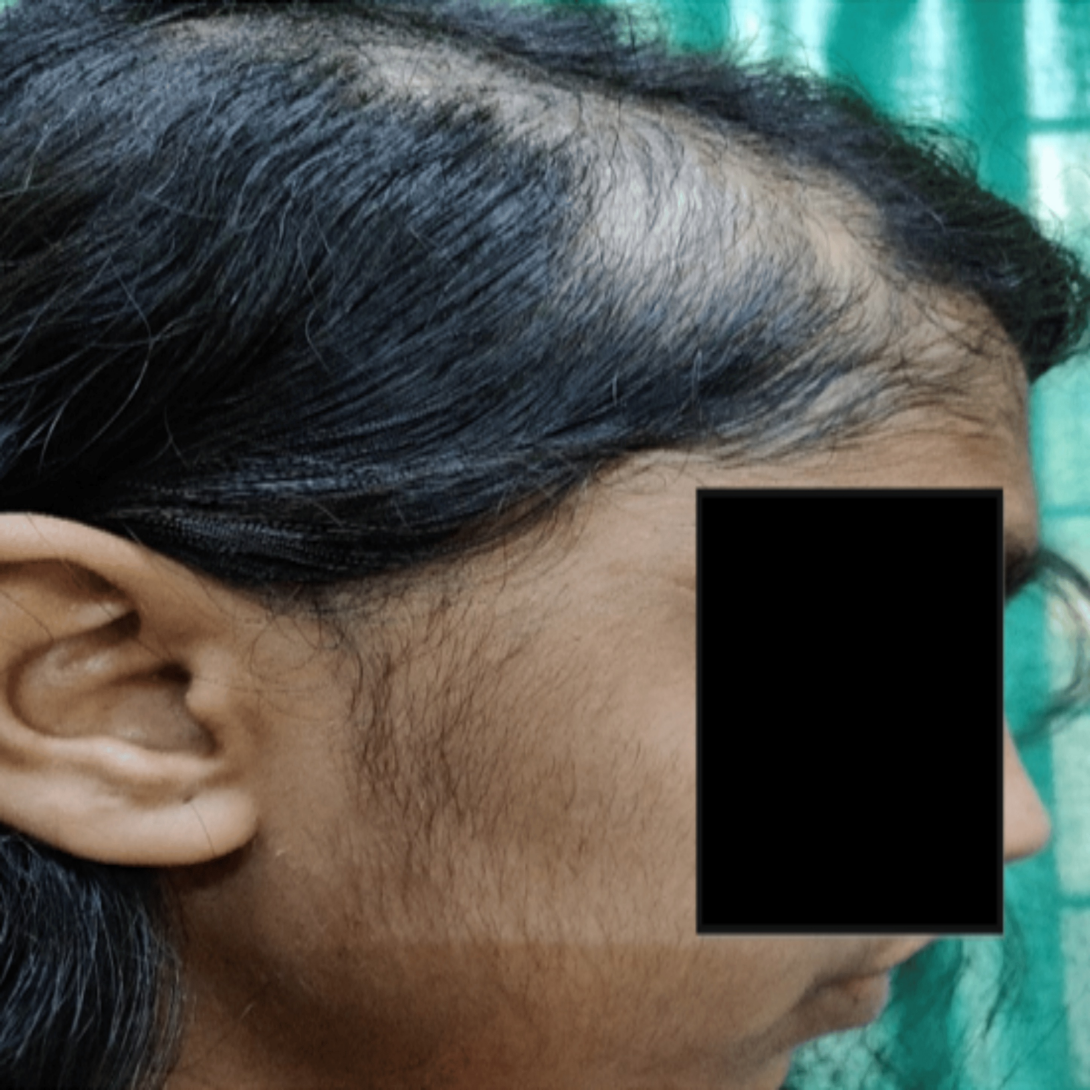
Hypertrichosis on the cheek area of a 16-year-old female due to cyclosporine therapy

Adverse effects to the therapy were noted in 23 (76.6%) partcipants in the cyclosporine group whereas in the betamethasone minipulse therapy group, 16 (53.3%) participants exhibited at least one side effect, with the same individual exhibiting multiple side effects at the same time due to the therapy, as detailed in Table 6. In Group A, gastrointestinal symptoms were the most common side effect seen in 13 (43.3%) participants, followed by hypertension in four (13.3%), hypertrichosis in three (10%), weight gain in two (6.7%), and headache/dizziness in one (3.3%) participant. In Group B, the most common side effect was weight gain, which was seen in nine (30%) participants, followed by mooning of face in seven (23.3%), acneiform eruption in four (13.3%), gastrointestinal symptoms in three (10%), headache/dizziness in three (10%), and skin atrophy in one (3.3%) participant, as detailed in Table 7. Figure 4 and Figure 5 show hypertrichosis caused due to cyclosporine therapy in females aged 8 and 16 years, respectively. No statistically significant difference in the incidence of side effects was observed between the groups.

**Table 6:** Distribution of patients according to occurrence of adverse effects Comparison of the occurrence of adverse effects between Group A and Group B. Chi-square test was used for statistical analysis. A p-value < 0.05 was considered statistically significant.

Adverse Effects	Group A		Group B		Chi-square value	p-value
							N	%	N	%
	Yes	23	76.66%	16			53.33%	3.5898	0.0581
No	7	23.33%	14	46.66%					
Total	30	100%	30	100%					

**Table 7:** : Distribution of patients according to the various side effects in each group Note: In the betamethasone mini-pulse group, a single individual suffered from multiple side effects.

Side Effects	Group A		Group B	
	N	%	N	%
Gastrointestinal symptoms	13	43.30%	3	10%
Hypertension	4	13.30%	0	-
Hypertrichosis	3	10%	0	-
Weight gain	2	6.70%	9	30%
Headache/dizziness	1	3.30%	3	10%
Mooning of face	0	-	7	23.30%
Acneiform eruption	0	-	4	13.30%
Skin atrophy	0	-	1	3.30%

Follow-up

The first month post-treatment follow-up showed the following: in Group A, no recurrence in five (16.7%) patients, recurrence noted in three (10%), and 16 (53.3%) patients improved but with sustained hair loss. There was no improvement at all in six (20%) patients; whereas in Group B, seven (23.3%) patients did not have any recurrence, there was recurrence in four (13.3%), and 12 (40%) patients improved, but with sustained hair loss. There was no improvement in seven (23.4%) patients. There was no significant difference between the groups (Table 8).

**Table 8:** Distribution of patients according to follow-up results at the first month post-treatment Comparison of follow-up results at the first month post-treatment in patients between Group A and Group B. Chi-square test was used for statistical analysis. A p-value < 0.05 was considered statistically significant.

Parameters		Group A		Group B		Chi-square value	p-value
		N	%	N	%		
Post-treatment 1 month	Cured/not recurred	5	16.70%	7	23.30%	1.124	0.771
	Recurred	3	10%	4	13.30%		
	Improved but sustaining hair loss	16	53.30%	12	40%		
	Not improved	6	20%	7	23.40%		
	Total	30	100%	30	100%		

At the second month of follow-up, in Group A, eight (26.6%) patients did not have any recurrence, recurrence was noted in two (6.7%) patients, and 15 (50%) patients improved but with sustained hair loss. There was no improvement in five (16.7%) patients. In group B, there was no recurrence in nine (30%) patients, recurrence was noted in two (6.7%) patients, and 13 (43.3%) patients improved but with sustained hair loss. There was no improvement in six (20%) patients. There was no significant difference between the two groups as per chi-square test (p>0.05) (Table 9).

**Table 9:** Distribution of patients according to follow-up results at the second month post-treatment Comparison of follow-up results at the second month post-treatment in patients between Group A and Group B. Chi-square test was used for statistical analysis. A p-value < 0.05 was considered statistically significant.

Parameters		Group A		Group B		Chi-square value	p-value
		N	%	N	%		
Post-treatment 2 months	Cured/not recurred	8	26.60%	9	30%	0.2926	0.95
	Recurred	2	6.70%	2	6.70%		
	Improved but sustaining hair loss	15	50%	13	43.30%		
	Not improved	5	16.70%	6	20%		
	Total	30	100%	30	100%		

At the third month of follow-up, 12 (40%) patients in Group A did not have any recurrence, recurrence was noted in one (3.3%) patient, and 13 (43.4%) improved but with sustained hair loss. There was no improvement in four (13.3%) patients. In Group B, 11 (36.7%) patients did not have any recurrence, recurrence was noted in one (3.3%) patient, and 12 (40%) patients improved but with sustained hair loss. There was no improvement in six (20%) patients. There was no significant difference between the groups as per chi-square test (p>0.05) (Table 10).

**Table 10:** Distribution of patients according to follow-up results at the third month post-treatment Comparison of follow-up results at the third month post-treatment in patients between Group A and Group B. Chi-square test was used for statistical analysis. A p-value < 0.05 was considered statistically significant.

Parameters		Group A		Group B		Chi-square value	p-value
		N	%	N	%		
Post-treatment 3 months	Cured/not recurred	12	40%	11	36.70%	0.4834	0.922
	Recurred	1	3.30%	1	3.30%		
	Improved but sustaining hair loss	13	43.40%	12	40%		
	Not improved	4	13.30%	6	20%		
	Total	30	100%	30	100%		

## Discussion

Although alopecia areata (AA) is a self-limiting condition where most cases tend to get cured spontaneously within a span of 1-2 years, most patients are anxious and seek an early cure because of severe cosmetic disfiguration and the stigma associated with it in society [1]. Even though many modalities of treatment are available for AA, including oral mini-pulse therapy of betamethasone and cyclosporine, there is a paucity of comparative clinical trial information on their efficacy and safety.

Therefore, this randomized, controlled, parallel-group clinical trial was designed to investigate the safety and efficacy of oral cyclosporine versus oral betamethasone mini-pulse therapy in patients with AA of different grades and clinical types in India.

The response rate was 16 (53.3%) in the cyclosporine group and 10 (33.3%) in the betamethasone mini-pulse group. This observation is similar to a study done by Yong Hyun Jang et al., where they found that the response rate was 54.9% in the cyclosporine group and 37.8% in the betamethasone mini-pulse group [9].

Among patients taking oral cyclosporine, 30% of the patients with severe AA (≥50% scalp hair loss, AT, and AU) responded to the treatment similar to a study done by Vivien Wai Yun Lai et al., where they found that 31.3% of participants with moderate to severe alopecia areata treated with oral cyclosporine achieved a response at the end of 3 months [10].

Amongst our patients who received oral betamethasone mini-pulse, we observed that only 10% of the patients with severe alopecia areata responded to the treatment at the end of 3 months. This finding is much inferior to that observed by YH Jang et al., where 33.3% of patients with severe alopecia areata responded to oral betamethasone mini-pulse therapy in 3 months [9].

Another study done by Khaitan et al. showed that 43.7% of patients with severe alopecia areata responded excellently, and 31.2% of patients showed a good response after 6 months of oral betamethasone therapy [11]. This finding could have been similar to our study had the oral betamethasone mini-pulse therapy been continued till 6 months in our study.

According to self-assessments of treatment responses, most of the cyclosporine group rated their hair regrowth as excellent or good (73.3%) while 26.7% rated it as fair or poor. In the betamethasone mini-pulse group, excellent or good hair regrowth was observed in 43.3% of patients, while 56.7% were rated as fair or poor. This difference is statistically significant (p<0.05). This observation is comparable with a study done by Yong Hyun Jang et al., in which on patient self-assessments, 70.6% of patients in the cyclosporine group and 43.2% of patients in the betamethasone mini-pulse group rated their hair regrowth as excellent or good [9].

Adverse effects to the therapy were noted in 76.6% (23/30) of patients in the cyclosporine group. All of them were of mild nature, except in four patients who developed mild hypertension at the end of three months of therapy. Most of the other adverse effects were reversible after completion of the therapy, and those who developed hypertension could be managed with antihypertensives. This observation is similar to a study done by Vivien Wai Yun Lai et al., where 83% of the participants reported various adverse events during the trial [10].

With cyclosporine therapy, the most common adverse effect observed was gastrointestinal symptoms, which were seen in 43.3% of cases, followed by hypertension (13.3%), hypertrichosis (10%), weight gain (6.7%), and headache/dizziness (3.3%). This observation is similar to a study done by Yong Hyun Jang et al. [9].

In the betamethasone mini-pulse therapy group, 53.3% (16/30) of patients exhibited at least one side effect, with the same individual exhibiting multiple side effects at the same time due to the therapy, and 14 individuals did not suffer from any side effects. This observation is comparable with a study done by JW Jang et al., where 58.7% (27/46) of patients exhibited at least one side effect due to betamethasone mini-pulse therapy [12].

In the oral betamethasone mini-pulse regimen group, the most common side effect was weight gain (30%), followed by mooning of face (23.3%), acneiform eruption (13.3%), gastrointestinal symptoms (10%), headache/dizziness (10%), and skin atrophy (3.3%). Similar side effects were also observed by Yong Hyun Jang et al. [9].

As the same individual in the oral betamethasone mini-pulse regimen group exhibited multiple side effects at the same time, the incidence of various side effects was higher in this group, even though the number of individuals suffering from adverse events was less as compared to that of the cyclosporine group. None of the side effects in either group were severe enough to stop the regimen and were managed with symptomatic treatments, and all were reversible after completion of therapy.

The follow-up results were almost similar to those observed in a study done by Yong Hyun Jang et al., where they followed up all cases monthly for a period of 3 months after the stoppage of therapy [9].

Based on the findings of this study, oral cyclosporine appears to be a more efficacious treatment option than oral betamethasone mini-pulse therapy for alopecia areata, demonstrating a significantly higher overall response rate and better patient-perceived hair regrowth, particularly in cases of severe alopecia. While cyclosporine was associated with a higher incidence of mostly mild adverse effects, the superior efficacy suggests it may be considered as a first-line treatment, especially for patients with more extensive hair loss. Our study can be considered unique in the context that this was a prospective study, in contrast to the retrospective ones done previously by other authors.

The following may be considered as the limitations of this study: being an open-label study without blinding, tapering off of the dose of oral betamethasone by 1 mg every week for the next 3-6 weeks following 3 months of intensive therapy, which coincided with the follow-up period of Group B patients, being a single-center study, and the trial having more subjective and observer-biased assessment parameters.

## Conclusions

Our research is a novel and distinctive addition to the field of dermatology, as we have conducted the first prospective study comparing the efficacy and safety of oral cyclosporine versus oral betamethasone mini-pulse therapy in the treatment of alopecia areata. To our knowledge, only retrospective studies comparing these two modalities are available in the literature.

In the present study, cyclosporine therapy shows more efficacy as compared to oral betamethasone mini-pulse therapy. However, a larger number of patients suffered from adverse effects in the cyclosporine group than in the oral betamethasone mini-pulse therapy group. Since in our study, cyclosporine was found to be more efficacious with no very serious or irreversible adverse effects, it may be considered among the first-line choices in the therapeutic armamentarium of dermatologists to treat alopecia areata. However, this needs to be substantiated by large-scale studies with a longer duration of therapy and larger sample sizes. Physicians can better tailor therapeutic approaches according to the patient's needs if they have a varied number of treatment alternatives in their armamentarium, thus improving outcomes and quality of life.
